# A Novel Strain-Based Method to Estimate Tire Conditions Using Fuzzy Logic for Intelligent Tires

**DOI:** 10.3390/s17020350

**Published:** 2017-02-10

**Authors:** Daniel Garcia-Pozuelo, Oluremi Olatunbosun, Jorge Yunta, Xiaoguang Yang, Vicente Diaz

**Affiliations:** 1 Mechanical Engineering Department, Universidad Carlos III de Madrid, Avd. De la Universidad, 28911 Madrid, Spain; dgramos@ing.uc3m.es (D.G.-P.); vdiaz@ing.uc3m.es (V.D.); 2 School of Mechanical Engineering, University of Birmingham, Edgbaston B15 2TT, UK; o.a.olatunbosun@bham.ac.uk; 3 Vanlead Rubber & Tire Research Institute, Wanli Tire Corporation Limited, Guangzhou 510425, China; neuasyang1@aliyun.com

**Keywords:** intelligent tires, strain gauges, traffic safety, tire conditions, fuzzy logic

## Abstract

The so-called intelligent tires are one of the most promising research fields for automotive engineers. These tires are equipped with sensors which provide information about vehicle dynamics. Up to now, the commercial intelligent tires only provide information about inflation pressure and their contribution to stability control systems is currently very limited. Nowadays one of the major problems for intelligent tire development is how to embed feasible and low cost sensors to obtain reliable information such as inflation pressure, vertical load or rolling speed. These parameters provide key information for vehicle dynamics characterization. In this paper, we propose a novel algorithm based on fuzzy logic to estimate the mentioned parameters by means of a single strain-based system. Experimental tests have been carried out in order to prove the suitability and durability of the proposed on-board strain sensor system, as well as its low cost advantages, and the accuracy of the obtained estimations by means of fuzzy logic.

## 1. Introduction

Tires have always been an area of interest to people and the transport industry as well, principally due to the impact they have on performance, comfort, fuel consumption and safety of vehicles. The last one has always been one of the reasons to develop an intelligent tire that is able to provide information about tire and road conditions. This intelligent tire would also be able to interact with different vehicle control systems to improve their performance as well as warning the driver of potential hazards. In this way, the vehicle would have an active safety system provided with reliable information about the tire working conditions. This information could be used by different control systems individually, such as Anti-lock Braking System (ABS), Traction Control System (TCS), Electronic Stability Control (ESC), Suspension Control System (SCS), etc.

Thus, an intelligent tire could help to prevent accidents and become a key part in vehicle safety systems. Hence, the intelligent tire concept would have the final aim of monitoring, in real time, the forces at the tire-road interface, contact patch length, friction coefficient, slip angle, road condition, and tire wear.

Regarding developments in “intelligent tires”, in 2002, the USA passed the Tread Act for Tire Pressure Monitoring System (TPMS) to warn the driver if a tire is significantly under-inflated [1]. Ten years later, the European Union also released its relevant legislation on TPMS. Although TPMS is a great achievement (and tires such as Nokian ITT or Goodyear Unisteel are good examples of its success), intelligent tire technology has bigger prospects than TPMS. The information that intelligent tires could generate, such as tire forces and friction coefficient, has the potential for improving the effectiveness of vehicle dynamics control systems.

**Figure 1:**
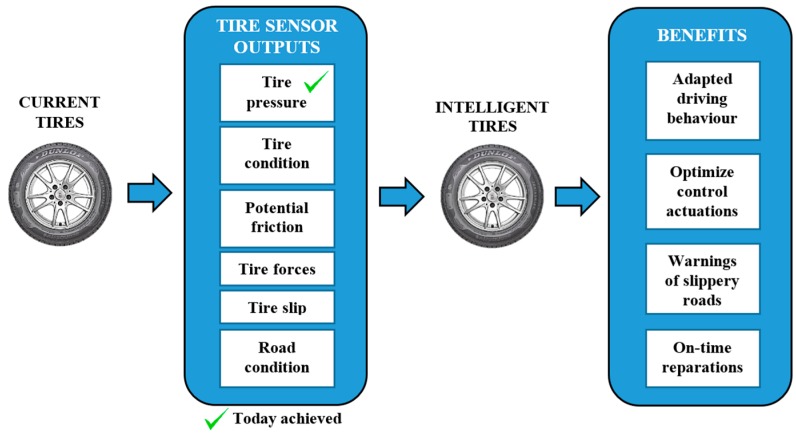
State-of-the-art in the use of tire-related information for vehicle control and driver information.

In addition to the TPMS, there are already a number of sensors providing information on vehicle dynamics such as yaw rate, longitudinal and lateral acceleration, differences in wheel rotation speeds and engine torque, etc. However, they have not yet been used to provide accurate information related to the tire-road contact (see Figure 1).

Due to tire-road contact features, which are nonlinear, it is difficult to apply the exact brake pedal force to stop the vehicle for the minimum distance without locking the wheels and, as a consequence, losing control of the vehicle which may result in a traffic accident unless ABS, TCS or ESC take action. In addition, this situation could be made worse when different road conditions are taken into account such as wet or muddy surface where friction is changing [2]. On the other hand, the grip limit is related mainly to the tire slip conditions and vertical load, this parameter being one of the most important for vehicle stability control systems, especially during cornering.

Although these systems perform well in a variety of situations, their performance can be improved if a real-time estimation of tire-road contact parameters is available. Some attempts with regard to the road condition and the real-time estimation of the tire-road contact parameters have been carried out. For instance, Gabbi et al. [3,4] developed a new measuring wheel and presented new chassis control strategies based on tire force measurements.

The influence of potential parameters such as inflation pressure, speed and vertical load on tire strain values, which can contribute to the estimation of tire behavior and provide information for vehicle systems and drivers, are analyzed herein. Some of these tire features have been studied in many works related to the intelligent tire. Overall, most of them are focused on measured deformation of the tire, such as strain, deflection, acceleration, etc. by means of different sensors with the same final objective: developing an intelligent tire system.

However, in order to install sensors in a tire, many problems have to be considered, such as the compatibility of the sensors with tire rubber properties (for instance, stiffness issues, data transmission or economic issues relating to the use of expensive sensors), but above all the main obstacle is to meet the power requirements of all the electronic components. An optimized material system that offers the prospect of a reduction of power for sensors implementation within battery-operated measurement systems was proposed by Yilmazoglu et al. [5]. In his interesting analysis of the feasibility of an onboard vibration energy harvesting system for a tire, Singh et al. [6] investigated various parameters, such as tire speed, vertical load, inflation pressure and road roughness conditions by measuring accelerations in real-time. Interesting results about tread acceleration measurements and wireless tire data transmission systems were also presented by Negrus et al. [7]. A magnetic sensor was developed by Miyoshi et al. [8], which can measure features such as radial and circumferential strain on the tread in order to relate the strain data with the tire forces. Stelzer et al. [9] developed a Surface Acoustic Wave (SAW) sensor that is capable of measuring some mechanical features such as pressure, temperature and deformation. This sensor is also useful to measure the road friction coefficient which could be of great help for vehicle control systems and driver-assistance systems such as ABS or Electronic Stability Control (ESC) [10].

In relation to the tire-road friction coefficient, several attempts to estimate it have been made [11,12,13]. Hong et al. [14] proposed an algorithm that first determines the contact patch by means of a radial acceleration profile and then the lateral acceleration profile of the tire–road contact patch is used to estimate the friction coefficient. A device capable of estimating tire-road contact forces was developed by Cheli et al. [15]. It also assessed the reliability of the measurements by comparing the longitudinal/lateral forces and the product of the measured longitudinal/lateral accelerations times the vehicle mass. Tuononen [16,17] has carried out several studies to measure tire carcass deflections by means of an optical sensor to provide estimations about the vehicle state. Successful results were obtained by Cullen et al. [18], who used segmented capacitance rings to measure the inflation pressure of the tire. In fact, this sensor is also used to measure deformation or strain of the tire in some different applications. Finally, Magori et al. [19] installed an ultrasonic sensor on the base of a wheel rim to monitor the deformation, temperature and other contact patch features.

Past researches related to intelligent tires indicate that strain sensors meet the requirements to achieve an advanced intelligent tire system by means of strain measurement [20,21]. In addition, strain sensors are less expensive than other sensors, reliable and accurate enough to measure strain data under dynamic conditions.

The aim of this paper is to corroborate that the strain gauges are suitable sensors to provide useful information about the working conditions of tires. The experiments have been carried out by means of strain sensor equipment and an indoor tire test rig. These devices were selected to study the tire tread behavior based on strain measurements. Based on experimental data, a method using fuzzy logic to estimate vertical load and rolling speed is described. This paper is a continuation of the experiments carried out to measure tire dynamic strain on the inner surface of the tire [21].

## 2. Materials and Methods

### 2.1. Strain-Based Experiment Setup

**Figure 2:**
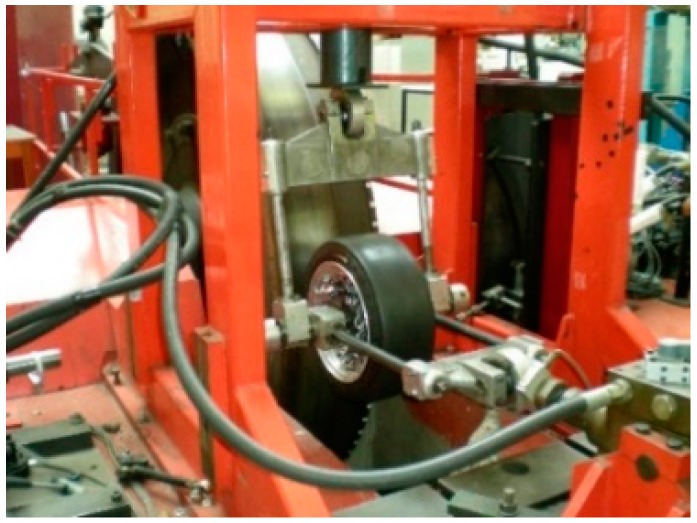
Indoor tire test rig.

The test system used for the experiments is an indoor tire test rig which makes it possible to vary speed and vertical load. Although it has not been carried out in this experiment, the tire test rig also allows one to change the surface by installing rough sheets on the drum. The experiment setup (including the indoor tire test rig equipped with tire) is shown in Figure 2.

The main feature of this device is that the surface is curved. Although it might be thought that this fact implies a disadvantage due to the conditions not being wholly realistic, in order to check if the curvature of the tire test rig induces errors or modifies the measured data, some of the tests have also been carried out on a flat surface, concluding that it is not an obstacle. As the variations in results were not significant, the disturbances due to the surface curvature could be considered insignificant.

Regarding strain sensors, from our literature review about strain-based intelligent tire systems, it was found that the strains are often measured at the tire tread in relevant researches [21,22], although they have been also installed in other elements like bearings [23,24]. These reveal that a strain sensor placed on the inner surface of the tire can provide useful information about the tire forces [20]. The strain sensors used in this study are also located at the inner liner surface of the tire tread. However, it should be highlighted that measurements can be attenuated because of the tread thickness in comparison with measurements from the outer tire tread.

**Figure 3:**
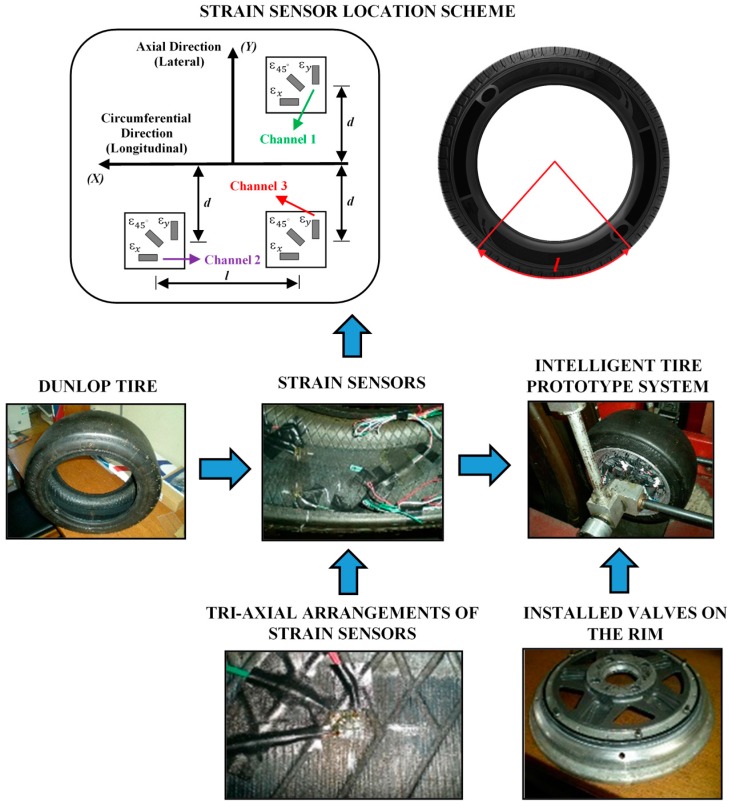
Strain gauges configuration.

These strain gauges, which are applicable for large strain measurements, have been attached at different points of the inner surface of the tire in order to measure lateral (ε_y2_—Channel 1 and ε_y1_—Channel 3) and longitudinal (ε_x_—Channel 2) strains, as shown in Figure 3. It can be assumed that the stiffness of the strain gauge doesn’t cause local stiffening effect. The strain gauge’s length is 2 mm with gauge resistance 120 Ω. The resolution of the strain measurement is 0.001 με.

First of all, it is important to show the strain gauges’ position. There are three multiaxial strain gauges, two of them in the same cross section and the third one separated by 123.75° of angular rotation, as shown in Figure 3. Figure 3 also shows the support installation of the rectangular rosette strain sensors on the inner surface of the tire. It was made using a manufacturer recommended adhesive for rubber materials.

Three new valves were installed on the rim and the lead wires of the strain sensors were packed and routed through the valves to the outside of the tire (Figure 3). They were sealed to ensure that the air did not leak through the valves’ holes. The rim and the tire were sealed with adhesive and the tire was inflated to the desired inflation pressure. After that, it was left for 72 h in order to check the seal reliability. These three new valves allow the connection between strain sensors and data acquisition system.

**Figure 4:**
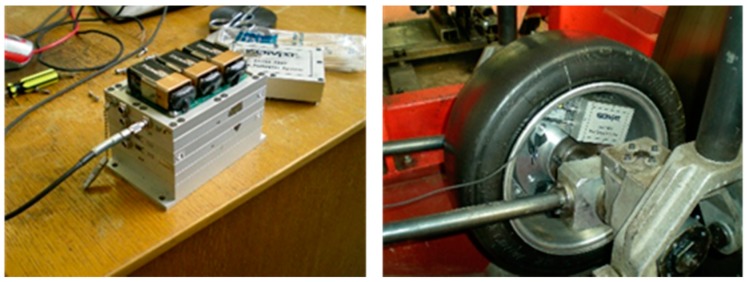
SoMat^®^ 2000 installed in the tire.

In this work, a SoMat^®^ 2000 Field Computer (Somat Corporation, Urbana, IL, USA) was used as a data acquisition system. This device is designed for portable data collection in several test environments. The hardware of the system consists of a Processor module, which has the microprocessor data acquisition system, and a Power/Communication module, equipped with batteries, as shown in Figure 4.

SoMat^®^ 2000 has one Wheatstone strain bridge for each strain sensor, which can also be used in a quarter, half or full bridge. The SoMat^®^ 2000 Field Computer was driven by Test Control Software for Windows (WinTCS). When a test starts, the data acquisition module (i.e., the strain gauge module) is connected to the strain sensors. After that, the user can download the test specification, designed using WinTCS, from a computer to the SoMat^®^ 2000 Field Computer to initialize and run it. By sampling the electrical analogue signals, SoMat^®^ 2000 can store and manipulate digital data as bytes of binary digits. The SoMat^®^ 2000 Field Computer can provide a sampling frequency from 0.0005 to 5000 Hz, which ensures that the test strain data resolution is adequate to monitor enough strain points per tire revolution.

It should be pointed out that many factors could influence the performance of the data acquisition system, which could be divided into two categories: properties of the data acquisition system and the tire properties (such as tire radius or rolling speed). Regarding properties of the data acquisition, it is obvious that a large number of data channels would extend the amount of the data acquired, and as a consequence, a large memory space would be required and the available test time would decrease. In this study, the sampling frequency was set to be 1000 Hz.

For the experiments, the DUNLOP SP SPORT 175/505 R13 (tubeless) slick radial tire was selected. This type of tire, which normally operates at low vertical loads and inflation pressures, is used for the Formula SAE. The speed used in this work was limited at 50 km/h, which is suitable for the sampling frequency used. In addition, the average speed in the circuits of the Formula Student is usually under that speed. The working range of the SoMat^®^ 2000 strain gauge module used was between −5000 and 5000 με.

The experiments carried out consisted of studying the tire strain behavior by changing test conditions in order to determine which parameter of the tire (inflation pressure, vertical load or speed) is the most influential and which measurement direction provides the most useful information about tire working conditions. It can be done by analyzing measurements from strain sensors, which can be downloaded to a computer after tests. Once it was known how strain values for different working conditions are generated, a fuzzy logic controller to estimate the vertical load has been developed.

### 2.2. Test Conditions

The operational range of parameters used for the straight line rolling conditions are:

- Tire inflation pressure: 0.8 bar–1.4 bar;
- Tire preload: 250 N–1000 N;
- Tire speed: 10 km/h–50 km/h;
- Tire slip angle: 0°;

## 3. Results

### 3.1. Experimental Data Analysis

The experimental data analysis allows us to know what information could be extracted from strain measurements. Despite the fact that only strain sensors have been used in this study, it should be highlighted that some of these tire operating conditions could be measured by different sensors, which allow the evaluation of the information provided by strain gauges, such as the inflation pressure, the time variation, the robustness, etc. Throughout this study, the accuracy as well as the robustness of the strain gauges are demonstrated, on both flat and curved surfaces. Specifically, the tests have been carried out for over 50 h and obtained data have shown very high repeatability, providing accurate results that can be used for different studies. As it has already been discussed, this type of sensors has also been selected because they are cheaper than other sensors such as accelerometers or optical sensors, providing similar results for the measurement of different features. In addition, as they are installed on the inner surface of the tire, the probability that they get damaged is low. However, due to strain sensors’ dimensions, its integration in the structure of the tire during the manufacturing process thereof would increase the robustness of the strain sensors as well as the time they can last.

Preliminary measurements of tire dynamic strain on the inner surface of the tire were performed under tire steady state and straight line rolling conditions. Before carrying out the test, the measuring system was calibrated and zeroed, checking that some factors like temperature, which could affect some measures such as the properties of the tire and the operation of the sensors, do not affect the data acquisition by ensuring that they were very similar for all the tests.

In straight line rolling conditions it is possible to study and understand the influence of several parameters on tire strains. Throughout different tests, it has been possible to identify the most influential parameter in strain values. Moreover, it is feasible to ensure the proper operation of the strain gauges in spite of changing the working conditions (either increase the speed, inflation pressure or vertical load).

**Figure 5:**
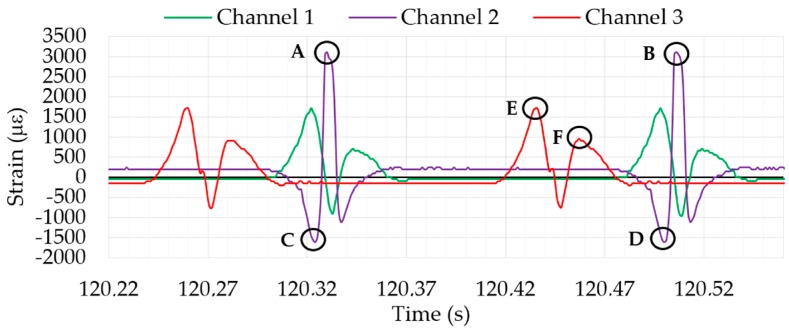
Example of strain data collected.

The graph shown in Figure 5 exhibits two peaks per revolution for each channel. For Channel 2, the distance between A and B represents the time it takes the wheel to complete a full revolution. Points A and B also represent the maximum tensile strain values recorded by Channel 2 when the sensor starts passing through the tire contact patch. Conversely, points C and D represent the maximum compressive strain values recorded by Channel 2 through the same process. It should be highlighted that the strain data measured from a rolling tire and the strain curve shape presented in Figure 5 change depending on tire working conditions (i.e., whether the tire is under braking or cornering efforts) as well as the tire structure parameters. Thereby, it is possible to collect the maximum tensile and compressive strain values for each channel and analyze which of the three working conditions (pressure, speed and vertical load) is the most influential in each one. The test data shown in Figure 5 were obtained for the following tire conditions: 0.8 bar inflation pressure, 30 km/h speed, and 750 N vertical load, at 0 degree slip angle. The strain curves conformed to the expected characteristics and there was good repeatability in each test.

As the distance between A and B represents the time that the tire spent in completing one revolution, there are 360° between these points, enabling the angular speed of the wheel to be obtained from the period. In this way, the shaft encoders (which provide information about the angular speed of the wheel) could be saved, because the intelligent tire could provide the same data regarding the wheel revolutions.

The variation in the strain data collected may also be useful information to identify the variation of tire working conditions. Since these strains are generated due to the corresponding efforts, especially those which occur in the tire-road contact patch, their characteristics have provided a significant role in the estimation of tire dynamic behavior, tire tread wear and other characteristics [25]. For instance, tensile strain peaks E and F (see Figure 5) change when the tire is involved in a braking/traction process [26,27]. In this aspect, the experimental data show promising results. They confirm that the tensile strain peak E increases while F decreases when tire is braking, and vice versa for tire traction. However, this part of the study is currently being developed and requires a specific analysis.

**Figure 6:**
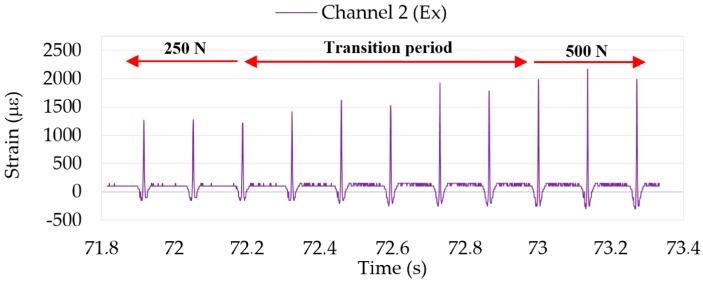
Strain data changes in Channel 2 for different vertical loads.

Finally, as the indoor test rig allows different parameters to be changed without stopping the intelligent tire prototype, the study of the changes in the shape of the curves is the fundamental part of the study. For instance, Figure 6 shows the transition period while vertical load is being changed. Thereby, by analyzing data changes it would be possible to achieve the ultimate goal: the estimation and prediction of the potential adherence in real-time.

### 3.2. Influence of Speed

**Figure 7:**
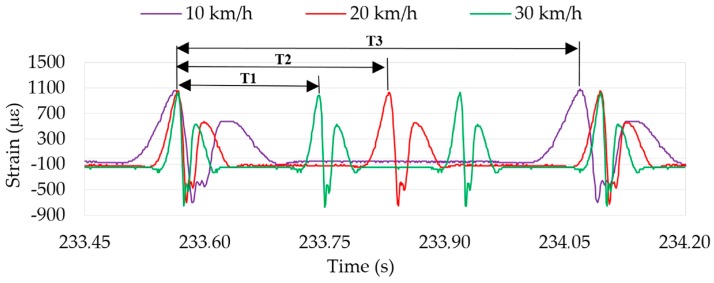
Speed influence in the period.

The first experiments were carried out varying speeds with different vertical loads and inflation pressures. Deformation curves at 10, 20 and 30 km/h from the first maximum tensile strain point have been overlaid in Figure 7. It shows not only that, as might be expected, the wheel period decreases as speed increases, but also the difference in the curve width (i.e., the contact patch length) due to the time the strain sensor spent in passing through the contact patch, in other words, the width between two consecutive maximum tensile strain points, approximately.

**Figure 8:**
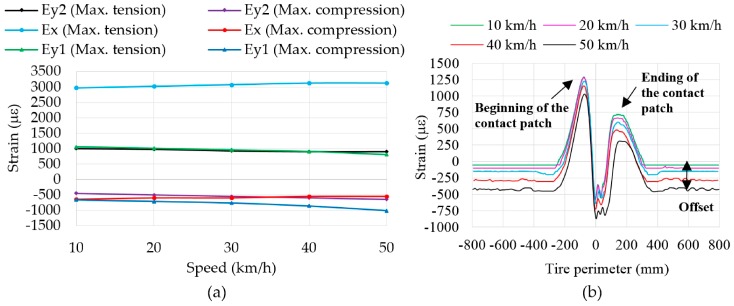
(**a**) Influence of speed in maximum strain values; (**b**) Comparison of curves at different speeds in ε_y2_ at 1 bar and 750 N.

However, little changes occur in the curves’ shape (overall near to the maximum tensile and compressive strain points) can also be caused by the increase in speed, because for the same sampling frequency the number of acquired data points per revolution is lower. Due to this fact, data processing has been carried out in order to obtain the strain value for each point of the tire perimeter instead of as a function of time, as it is shown in Figure 8b.

Figure 8a shows that maximum tensile strain values in ε_y1_ and ε_y2_ decrease as speed increases, in contrast to ε_x_. In addition, it is noted that, while the amplitudes (the difference between maximum tension and compression values) of ε_y1_ and ε_y2_ remain practically constant, the amplitude measured by the Channel 2 (ε_x_) increases considerably, overall from 20 km/h.

The difference between ε_y1_ and ε_y2_ data can be influenced by the curvature of the inner surface of the tire, which is related to the camber angle. Despite the sensors are placed symmetrically relative to the center line of the tread, the small variation between the values of ε_y1_ and ε_y2_ could provide information about this aspect.

Thus, it is verified that there are significant changes in strain values due to speed increments. It is also noted that when the sensor is away from the contact patch (i.e., the intervals (−800, −300) mm and (400, 800) mm, Figure 8b), strain sensors keep providing useful information about strain, particularly in compression, whose values increase as speed increases, due to the centrifugal force.

### 3.3. Influence of Pressure

**Figure 9:**
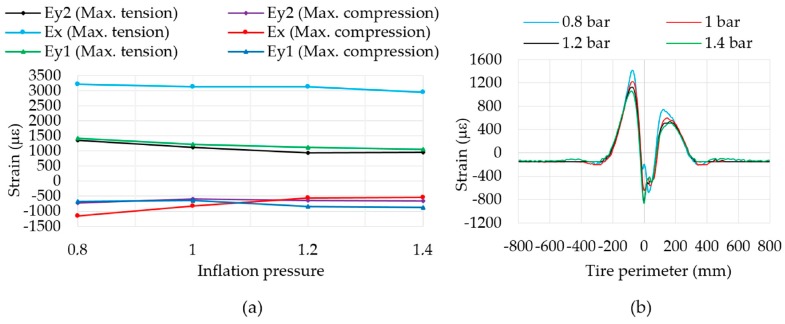
(**a**) Influence of pressure in maximum strain values; (**b**) Comparison of curves at different pressures in ε_y1_ at 30 km/h and 750 N.

In the experiments with pressure changes it is observed that when pressure increases, the stiffness of the tire also increases and, as a consequence, deformation decreases, as it is shown in Figure 9a. The results have confirmed that lateral deformation is stable. This corresponds to the fact that the shoulder area of the tire works harder than the tread in that direction.

Tensile strain values decrease for the three channels, while the maximum compressive strain value only decreases in ε_x_, therefore, the amplitude in longitudinal direction (ε_x_) decreases when the pressure increases (see Figure 9a). Curves at different pressures for ε_y1_ have been overlaid in Figure 9b. Note that there exists a direct proportional relation between the pressure increment and the increment in maximum compressive strain values due to the increment in the stiffness of the tire. As is well known, at low pressures the tire leans more on the shoulder of the tire than the central part of the tread, while if the pressure is high the tire leans excessively on the central part thereof. Thus, when the shape of the contact patch varies, changes in strain values in the central part of the contact patch (around to 0 mm) occur.

### 3.4. Influence of Vertical Load

**Figure 10:**
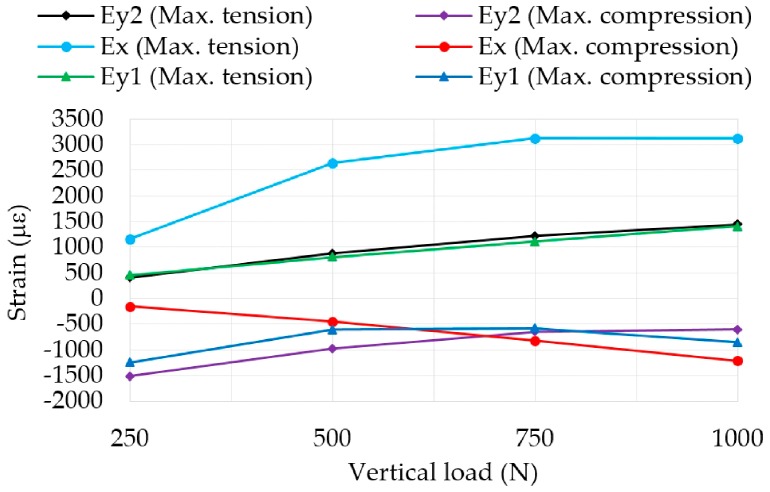
Influence of vertical load at 1 bar, 30 km/h.

Vertical load changes affect both tension and compression strains in the tire, as shown in Figure 10. In case of maximum tensile strain values, strains increase in both lateral and longitudinal directions. Nevertheless, maximum compression values only increase longitudinally (ε_x_).

**Figure 11:**
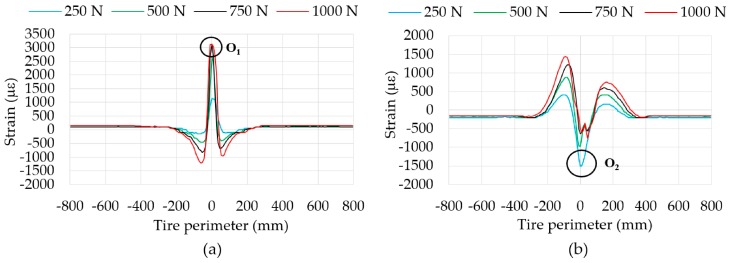
(**a**) Comparison of curves for different vertical loads in ε_x_ at 1 bar, 30 km/h; (**b**) Comparison of curves for different vertical loads in ε_y1_ at 1 bar, 30 km/h.

With the aim of developing the intelligent tire, it is especially interesting to observe from Figure 10 that tensile strain values increase linearly in the case of ε_y_. Thus, in order to measure changes in vertical load, it would be recommended to use sensors disposed laterally. Finally, Figure 10 shows that information provided by ε_y1_ and ε_y2_ is equivalent. Figure 11a,b shows curves for different vertical loads for ε_x_ and ε_y1_, respectively.

Contrary to pressure and speed changes, ε_x_ maximum tension values show significant differences for different vertical loads, however, they are not as linear as in the case of ε_y_. The data confirm that the reduction of maximum tensile strain values are directly related to the loss of grip, since the smaller the vertical load on the tire, the lower grip it can generate. In order to achieve good data and methods for predicting the available grip this specific part of the study is being developed currently.

Within the contact patch area, around the tire contact patch center (0 mm), fluctuations for ε_x_ strains (point O_1_ in Figure 11a) do not occur, contrary to ε_y1_ (point O_2_ in Figure 11b). For low pressures and loads, there is a slighter contact between the road and the 0 mm point (center of the contact patch).

## 4. Fuzzy Logic Implementation

### 4.1. Fuzzy Logic Framework

Fuzzy logic is a methodology which makes possible a decision-making process based on some initial parameters. The fuzzy logic framework is virtually built up in MATLAB (The Math-Works, Natick, MA, USA) by means of the fuzzy-logic toolbox and implemented in the simulation environment MATLAB/Simulink. Simulation results will be used to demonstrate the feasibility of the fuzzy logic to estimate vertical load and rolling speed values from strain data. In this Section, a brief overview of the generic framework of the fuzzy-logic inference system is given.

**Figure 12:**
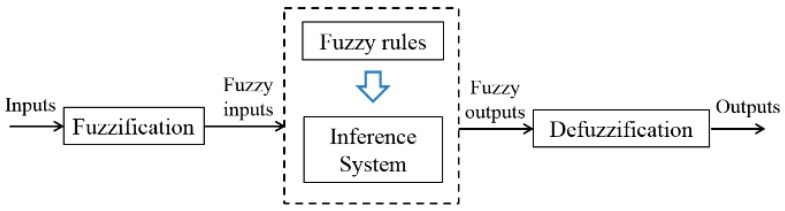
Scheme of a fuzzy system.

The procedure in which a Fuzzy-logic Inferential System (FIS) deduces the output values based on inputs can be explained into three steps (see Figure 12): fuzzification, fuzzy inference and deffuzification. A more detailed description of a fuzzy set is provided by Boada et al. [28].

In this particular work, the Mamdani inference, in which inputs are first fuzzified using membership functions, is used. This operator can be mathematically expressed as:

(1) $$f\left(\mu_{A}\left(x\right),\;\mu_{B}\left(y\right)\right)=\mu_{A}{\left(x\right)}^{\mu_{B}\left(y\right)}$$

**Figure 13:**
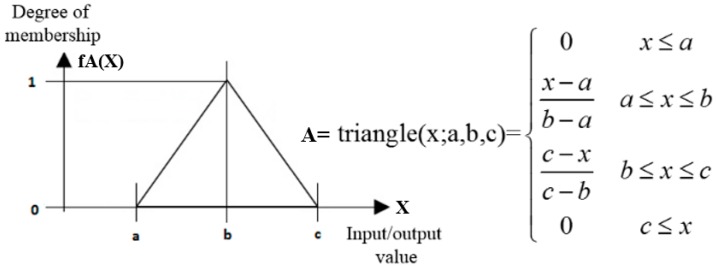
The membership functions for a generic variable.

In order to understand how it works, let’s assume *X* (the horizontal axis in Figure 13, either input or output values) is a space of points and the generic element of *X* is denoted by *x* (*X* = {*x*}) (see Figure 13). A membership function *fA*(*x*) characterizes a fuzzy set of *A* in *X* in which each point in *X* is a real number in the [0, 1] interval and the values of *fA*(*x*) at x represent the degree of membership of *x* in *A*. If the value of *fA*(x) is closer to 1, it means x has a higher degree of membership in *A*. Finally, $\mu_{A}\left(x\right)$ indicates the membership function of *x*. For simplicity, this study used triangular membership functions. The distribution can be mathematically expressed as shown in Figure 13.

Once the membership functions are defined, the fuzzified inputs are combined according to fuzzy rules. As every value either in the inputs ball or the outputs domain has a belonging degree to each of the categories, the response is automatically determined depending on this value, during the so-called defuzzification. The defuzzified output is obtained by the centroid method:

(2) $$z^{*}=\frac{\int \mu_{i}\left(z\right)\cdot z\cdot dz}{\int \mu_{i}\left(z\right)\cdot dz}$$

where $\mu_{i}\left(z\right)$ is the aggregated membership function and *z* is the output variable.

In this particular work, the fuzzy logic has been used to estimate vertical load and rolling speed. As it is well known, when load transfer is increased, the effective grip is reduced [29]. So, the knowledge about how load transfer behaves give us a good approach for estimating the potential grip.

Three inputs have been considered for its fuzzification (see Figure 12): the maximum tension peaks in ε_y1_ at the beginning and ending of the contact patch (input 1 and 2, respectively) and the offset values (input 3), which are indicated in Figure 8b. Since the offset value only varies significantly with speed (see Figure 8b, Figure 9b and Figure 11b) in comparison with pressure and vertical load, it has been used as input to estimate more accurately the values of the speed by fuzzy logic. As said above, the rolling speed could also be determined by the period from peak to peak (see Figure 7). However, since it depends on the resolution and the sampling frequency of the data acquisition system, in this work the rolling speed has also been estimated by fuzzy logic.

**Figure 14:**
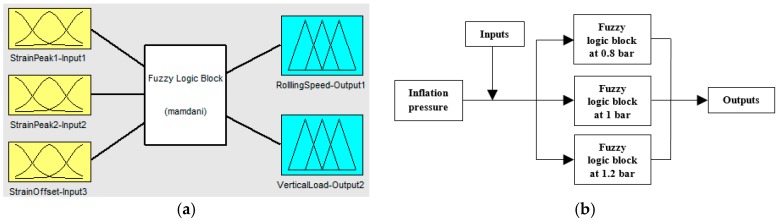
(**a**) Fuzzy Logic toolbar interface; (**b**) Working scheme used to obtain outputs.

To sum up, the parameters to be estimated (outputs) will be the rolling speed (output 1) and the vertical load (output 2) while the inputs are the mentioned strain data. Figure 14a shows schematically the Fuzzy Logic toolbox interface as well as the inputs and outputs that have been taken into account.

Finally, despite the fact that inflation pressure (P) could be a variable to be determined based on strain data, it is not a variable which estimation implies today is an obstacle, since it is correctly measured by TPMS, so in this simulation work it has been taken as a known parameter, so that three fuzzy logic blocks have been developed for 0.8, 1 and 1.2 bar, since 1 bar is considered the nominal pressure of the tire used in this work. In this way, as inflation pressure does not change from instant to instant (unless a puncture happens), once it has been measured, the corresponding fuzzy logic block can be used (see Figure 14b).

As can be deduced, the definition of the Fuzzy rules between input and output variables as well as the membership functions are of essential importance for development of the Fuzzy Logic Block.

The fuzzy rules used define the relationship between input and output variables, indicating the way vertical load behaves. There were used a set of 232 if→then rules like this:

(3) $$\mathrm{if}\left(\begin{array}{c} \mathrm{input}\;1\;\mathrm{is}\;I1\;\mathrm{and}\;\mathrm{input}\;2\;\mathrm{is}\;I2\\ \mathrm{and}\;\mathrm{input}\;3\;\mathrm{is}\;I3 \end{array}\right)\;\mathrm{then}\left(\mathrm{output}\;1\;\mathrm{is}\;O1\;\mathrm{and}\;\mathrm{output}\;2\;\mathrm{is}\;O2\right)$$

where I1, I2, I3, O1 and O2 are values of these variables.

**Figure 15:**
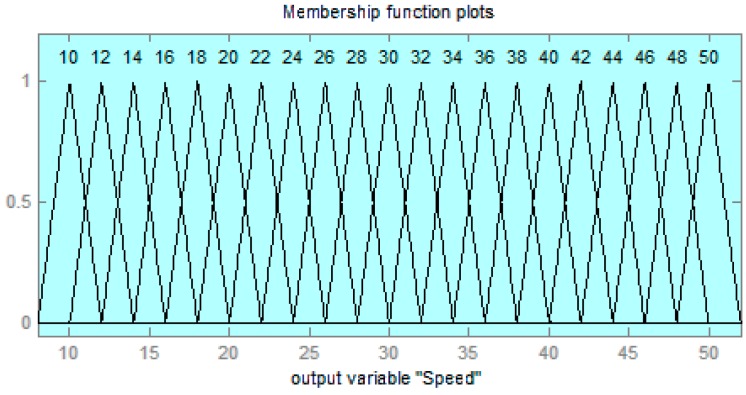
The membership functions plots for speed variable.

As shown in Figure 15, triangular functions have been used. By way of example, it shows the manner in which the speed was broken down into 21 levels from 0 to 55 km/h:

Table 1 summarizes the number of levels in which each variable of each fuzzy logic block has been broken down (see Figure 13) and the range of values they cover.

### 4.2. Simulation Results

In this Section, results obtained from the fuzzy-logic toolbar in comparison with experimental data at different pressures are shown. Before showing the results, it is important to point out that, as stated in Section 2.2, the experimental tests have been performed for loads every 250 N and speeds every 10 km/h. Because of this, in order to compare simulation results with intermediate experimental values and determine whether or not they keep good relation with the values that should be obtained, intermediate values by means of the regression curves were calculated. In this way, different simulations were carried out not only for the experimental data but also with other intermediate values.

**Figure 16:**
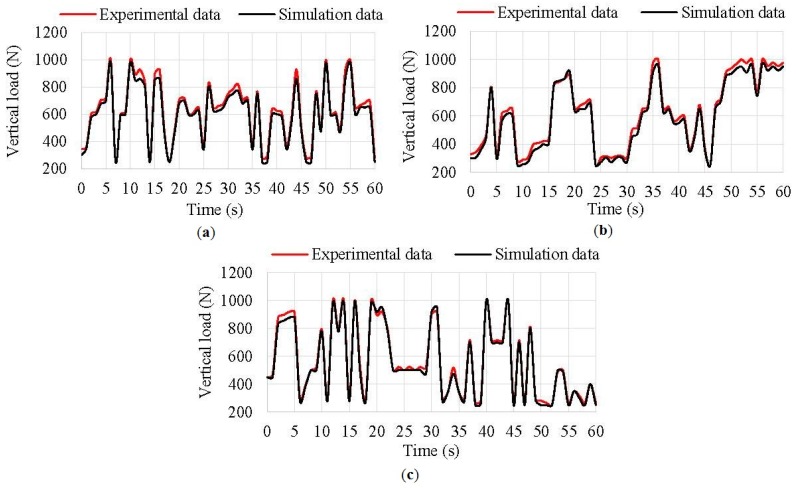
(**a**) Vertical load simulation results at 0.8 bar; (**b**) Vertical load simulation results at 1 bar; (**c**) Vertical load simulation results at 1.2 bar.

As inflation pressure does not change from time to time, it has not been changed during simulations. Figure 16 shows obtained simulation results using Fuzzy Logic versus experimental data.

The relationship between experimental data and simulation results is quite accurate for any inflation pressure. During the simulations, the average error was 6% at 0.8 bar, 6.2% at 1 bar and 3.15% at 1.2 bar. However, the average error reduced considerably taking only into account the known data set (i.e., data non-calculated by regression curves). As a result, curves from model and experimental curves practically overlapped. Thus, a more complete set of experimental data allows us to achieve better estimations.

**Figure 17:**
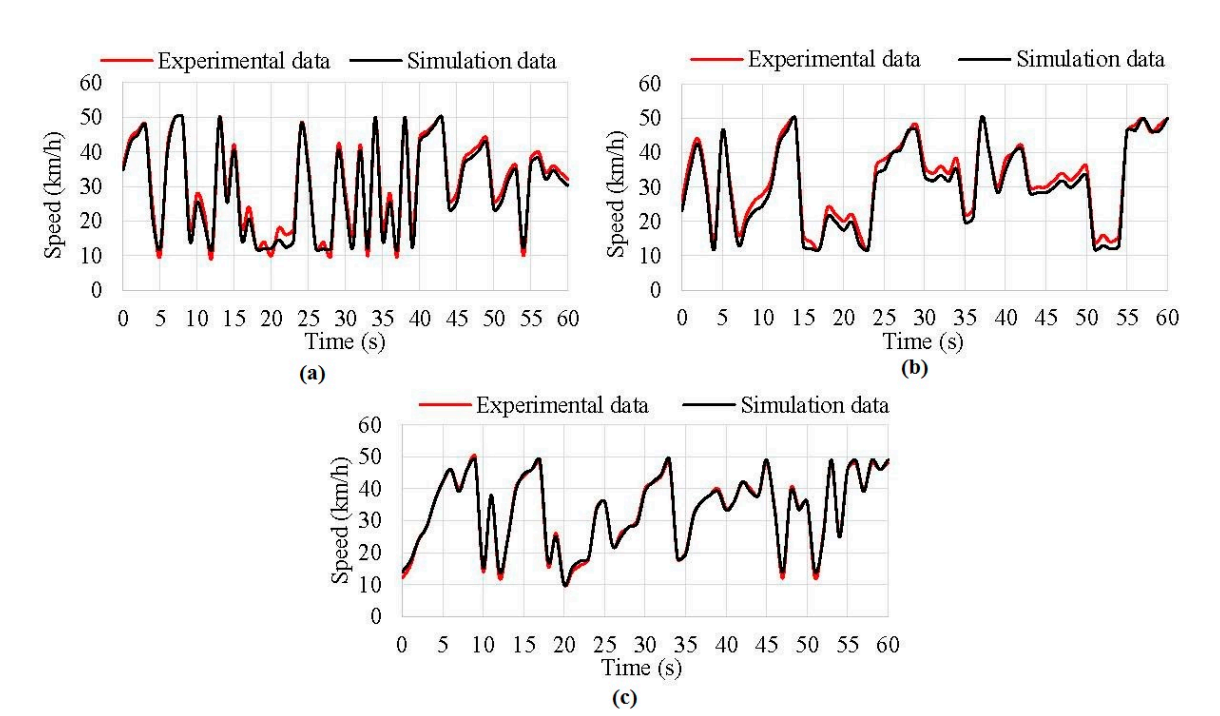
(**a**) Rolling speed simulation results at 0.8 bar; (**b**) Rolling speed simulation results at 1 bar; (**c**) Rolling speed simulation results at 1.2 bar.

Regarding simulation results for rolling speed, the obtained results show that the difference between experimental data and simulated data was under 2 km/h for any inflation pressure while the average error halved (lower than 3% in any situation) for the known data set. Figure 17 shows results of simulations at different pressures.

As can be observed, the curves practically overlapped each other, mainly at 1.2 bar. Thus, it is demonstrated that fuzzy logic can estimate the rolling speed from offset values.

It has to be noted that the variation in time of the values of the *x*-axis (Figure 16 and Figure 17) corresponds to each instance of the simulation, and not to a real case. Since speed and vertical load values have been estimated taking into account the experimental strain data at steady state, where acceleration and braking processes are not severe.

Finally, if a tire subjected to the same vertical load as the others is rolling at higher speed, it could be deduced that tire is sliding and must be braked. This information could be used by either ABS or TCS. In addition, together with tire acceleration and the vehicle dimensions, vertical load can be significantly useful to estimate many other parameters of the vehicle dynamic behaviour such as the friction coefficient or load transfer, which are still a challenge to be achieved.

## 5. Discussion

In this paper, tests related to straight line rolling conditions at steady state (without severe acceleration/braking processes) have been performed. The main equipment used has been: data acquisition system, indoor test rig, a tire and strain sensors.

The results presented in this study have shown that strain gauges are capable of accurate measurements of tire behavior under dynamic conditions. They have confirmed that the shape of the curve for each working condition give us interesting information and allows us make distinctions between these conditions. Next intelligent tire prototypes will take advantage of the results to improve some features such as weight, size, properties and sensor location in order to achieve a definitive intelligent tire system. Besides, the shape of the curves seems to be useful for other conditions (traction, braking or cornering). Current studies are showing promising results about this hypothesis.

When tire pressure increases, it clearly affects deformation mainly during the compression process. It is obvious that when inflation pressure increases, the tire rigidity also increases, and as a consequence, both the compression and tension deformation values during rolling are lower. Regarding experiments with different vertical loads, results indicate that both lateral and longitudinal strain sensors provide useful information about this aspect.

From Figure 8b, Figure 9b and Figure 11b it is observed around the tire contact patch center (0 mm) that there are fluctuations that hinder the information acquisition from significant peaks. These fluctuations are caused because they are within the interval (−10, 70) mm, which corresponds approximately to the contact patch length. Thus, providing the tire tread with strain gauges it would be possible to obtain information related to the contact patch, such as the contact patch length. It is also observed from these graphs that despite the strain gauges are placed in the middle of the contact patch and the strain gauges’ length is 2 mm, they are able to provide significant information about more than 500 mm of the tire perimeter, that is, more than one third of the whole tire perimeter. In this way, in order to obtain useful information from the whole perimeter, it would be enough to put in place strain gauges every 120°. However, for the purpose of studying and analyzing the tire behavior, it is enough to set up strain gauges at one point of the tire tread.

On the other hand, one of the most common problems in the tire’s performance is the delamination thereof, which consists of the separation of layers of rubber. This is not a trivial matter, because it can be the cause of stability problems, oversteer, or tire blowout. If several sensors were placed in the tread at different positions (for instance every 90°) and one of them measures the angular speed of the wheel wrongly relative to the other sensors, it could be possible to detect tire delamination problems around the area where the sensor is placed, because the delamination process can be caused by a hit, damaging the sensor and even the tire carcass.

Finally, the intelligent tire prototype used in this study has demonstrated that strain sensors are suitable for measuring dynamic behavior, mainly maximum tensile and compressive strains, which have been used to estimate the vertical load and rolling speed by means of fuzzy logic. However, taking into account the computational time, the fuzzy logic algorithm should be optimized to be capable of estimating conditions in real-time.

Simulation results have demonstrated the high potential of strain sensors to provide useful information from the characteristic points of strain curves. Although vertical load and rolling speed simulation results do not have a close relationship, it does not imply a hindrance, since vertical load not only change due to speed change, but also because of possible banks or potholes in the road surface as well as acceleration or braking processes with load transfer.

The ongoing future works are: to carry out test at different speeds and vertical loads in straight line and cornering conditions (varying the slip angle), corroborate the conclusions mentioned herein with other tires and improve the computational time of the fuzzy logic algorithm. In addition, the design of a wireless monitoring data system in order to estimate the potential grip in real-time is an objective to be achieved.

## Figures and Tables

**Table 1:** Number of levels and range of each variable.

	P	Input 1	Input 2	Input 3	Output 1	Output 2
**Levels**	0.8 bar	60	44	15	16	21
	1 bar	56	41	15	16	21
	1.2 bar	54	37	17	16	21
**Range**	0.8 bar	275 to 1800 με	−75 to 1050 με	−450 to 50 με	5 to 55 km/h	200 to 1050 N
	1 bar	175 to 1600 με	−100 to 950 με	−450 to −50 με	5 to 55 km/h	200 to 1050 N
	1.2 bar	150 to 1525 με	−100 to 825 με	−450 to 0 με	5 to 55 km/h	200 to 1050N

## References

[1] Transportation Recall Enhancement, Accountability, and Documentation (TREAD) Act. https://www.congress.gov/106/plaws/publ414/PLAW-106publ414.pdf.

[2] Austin L., Morrey D. (2000). Recent advances in antilock braking systems and traction control systems. Proc. Inst. Mech. Eng. Part D J. Automob. Eng..

[3] Gobbi M., Botero J., Mastinu G. (2008). Improving the active safety of road vehicles by sensing forces and moments at the wheels. Veh. Syst. Dyn..

[4] Gobbi M., Botero J., Mastinu G. (2009). Global chassis control by sensing forces/moments at the wheels. Int. J. Veh. Auton. Syst..

[5] Yilmazoglu O., Brandt M., Sigmund J., Genc E., Hartnagel H. (2001). Integrated InAs/GaSb 3D magnetic field sensors for “the intelligent tire”. Sens. Actuators A Phys..

[6] Singh K., Bedekar V., Taheri S., Priya S. (2012). Piezoelectric vibration energy harvesting system with an adaptive frequency tuning mechanism for intelligent tires. Mechatronics.

[7] Negrus E., Anghelache G., Sorohan S. (1998). Tire Radial Vibrations at High Speed of Rolling.

[8] Miyoshi A., Tsurita T., Kunii M. (2007). System and Method for Determining Tire Force. U.S. Patent.

[9] Stelzer A., Schimetta G., Reindl L., Weigel R. (2001). Wireless SAW Sensors for Surface and Subsurface Sensing Applications. Subsurf. Surf. Sens. Technol. Appl. III.

[10] Cyllik A., Strothjohann T., Scholl G. The Intelligent Tire—Applications of the Tread Sensor. Proceedings of the VDI BERICHTE.

[11] Breuler B., Eichhorn U., Roth J. Measurement of tyre/road friction ahead of the car and inside the tyre. Proceedings of the International Symposium Advanced Vehicle Control (AVEC).

[12] Müller S., Uchanski M., Hedrick K. (2003). Estimation of the Maximum Tire-Road Friction Coefficient. J. Dyn. Syst. Meas. Control.

[13] Lee C., Hedrick K., Yi K. (2004). Real-Time Slip-Based Estimation of Maximum Tire–Road Friction Coefficient. IEEE/ASME Trans. Mech..

[14] Hong S., Erdogan G., Hedrick K., Borrelli F. (2013). Tyre–road friction coefficient estimation based on tyre sensors and lateral tyre deflection: Modelling, simulations and experiments. Veh. Syst. Dyn..

[15] Cheli F., Braghin F., Brusarosco M., Mancosu F., Sabbioni E. (2011). Design and testing of an innovative measurement device for tyre–road contact forces. Mech. Syst. Signal Proc..

[16] Tuononen A. (2008). Optical position detection to measure tyre carcass deflections. Veh. Syst. Dyn..

[17] Tuononen A. (2009). Optical Position Detection to Measure Tire Carcass Deflections and Implementation for Vehicle State Estimation. Ph.D. Thesis.

[18] Cullen J., Arvanitis N., Lucas J., Al-Shamma’a A. (2002). In-field trials of a tyre pressure monitoring system based on segmented capacitance rings. Measurement.

[19] Magori V., Magori V.R., Seitz N. On-Line Determination of. Tire Deformation, a Novel Sensor Principle. Proceedings of the IEEE Ultrasonics Symposium.

[20] Morinaga H., Wakao Y., Hanatsuka Y., Kobayakawa A. The Possibility of Intelligent Tire (Technology of Contact Area Information Sensing). Proceedings of the 31st FISITA 2006 World Automotive Congress.

[21] Yang X., Olatunbosun O., Garcia-Pozuelo Ramos D., Bolarinwa E. (2013). Experimental Investigation of Tire Dynamic Strain Characteristics for Developing Strain-Based Intelligent Tire System. SAE Int. J. Passeng. Cars Mech. Syst..

[22] Matsuzaki R., Todoroki A. (2005). Wireless strain monitoring of tires using electrical capacitance changes with an oscillating circuit. Sens. Actuators A Phys..

[23] Kerst S., Shyrokau B., Holweg E. (2016). Reconstruction of wheel forces using an intelligent bearing. SAE Int. J. Passeng. Cars Mech. Syst..

[24] Kerst S., Shyrokau B., Holweg E. Wheel force measurement for vehicle dynamics control using an Intelligent Bearing. Proceedings of the 13th International Symposium on Advanced Vehicle Control (AVEC).

[25] Anghelache G., Moisescu R., Sorohan S., Bureţea D. (2011). Measuring system for investigation of tri-axial stress distribution across the tyre–road contact patch. Measurement.

[26] Matsuzaki R., Todoroki A. (2008). Intelligent Tires Based on Measurement of Tire Deformation. J. Solid Mech. Mater. Eng..

[27] Yang X., Olatunbosun O., Garcia-Pozuelo Ramos D., Bolarinwa E. (2015). FE-Based Tire Loading Estimation for Developing Strain-Based Intelligent Tire System.

[28] Boada B.L., Boada M.J.L., Díaz V. (2005). Fuzzy-logic applied to yaw moment control for vehicle stability. Veh. Syst. Dyn..

[29] Abe M., Manning W. (2009). Vehicle Handling Dynamics.

